# An Extendible Realism-Based Ontology for Kinship

**Published:** 2023

**Authors:** Michael Rabenberg, Anuwat Pengput, Werner Ceusters

**Affiliations:** 1 University at Buffalo, 77 Goodell Street, Buffalo NY, 14203, USA

**Keywords:** Kinship ontology, SNOMED CT, BFO2020

## Abstract

Adequately representing kinship relations is crucial for a variety of medical and biomedical applications. Several kinship ontologies have been proposed but none of them have been designed thus far in line with the Basic Formal Ontology. In this paper, we propose a novel kinship ontology that exhibits the following characteristics: (1) it is fully axiomatized in First Order Logic following the rules governing predicate formation as proposed in BFO2020-FOL, (2) it is modularized in 6 separate files written in the Common Logic Interface Format (CLIF) each one of which can be imported based on specific needs, (3) it provides bridging axioms to and from SNOMED CT, and (4) it contains an extra module with axioms which would not be literally true when phrased naively but are crafted in such a way that they highlight the unusual kinship relations they represent and can be used to generate alerts on possible data entry mistakes. We describe design considerations and challenges encountered.

## 1. Introduction

Ontologies are rarely developed completely from scratch these days, even if they don’t make use of any of the few upper ontologies that are around. Re-using ontologies, primarily parts thereof, has even become standard practice, specifically for ontologies rendered in OWL. However, it is not free of risks. Several issues with re-used content have been reported in BioPortal ontologies such as duplicated classes and object properties, inconsistent utilization of reused properties and redundant class hierarchies [1]. Tools such as ROBOT can detect certain syntactic errors in source ontologies used for import [2], and reasoners can detect logical errors within the boundaries of the logic they are designed for. Neither, however, can prevent the most common types of representation errors. One such type is caused by ‘*using OWL just as a syntax and ignoring its open-world semantics*’ [3, p11]. That is for instance the case with the majority of assertions of the form ‘*disease - has symptom - some - symptom*’ in the Disease Ontology [4]: the OWL semantics requires such statement to be true for ALL instances of the named disease, which is rarely the case. Although some recommend competency question-driven ontology authoring as a means to test the internal quality of an ontology before its release [5], doing so may result in ‘*tweaking the axioms so that the reasoner “gets the right answer” ignoring what else the axioms might entail*’ [3, p11]. Another form of tweaking is simply removing the axioms that otherwise would come with imported classes or object-properties, or creating axiom-less classes and object-properties with vague textual definitions and names that are slightly altered from what is found in reference ontologies.

Unfortunately, it is not only representation errors that hamper ontology reuse, but also differing perspectives in otherwise similar domains, as well as the contexts and applications for which they have been developed. Simply importing terms and axioms from domain- and application ontologies that are claimed to be internally coherent and consistent – even logically – is therefore not enough. In many cases, a thorough manual inspection on top of semi-automated procedures is therefore required. In this paper, we demonstrate the dilemmas that had to be faced in developing a realism-based kinship ontology for a specific use case while trying to maximally reuse what has already been developed. In Section 2, we explain realism-based ontology. In Section 3, we describe a use-case for our ontology, from which some of the relations in our ontology are derived. Section 4 contains a review of several extant kinship ontologies, including the one from which ours is primarily adapted. We describe our methodology in Section 5 and detail the results of our project in Section 6. Further discussion, including some of the more important design choices we made, appears in Section 7. Section 8 contains concluding remarks.

## 2. Realism-based ontology design

For something to be a realism-based ontology is for it (1) to be a representation of what is generically the case for a plurality of entities in reality, and (2) to be built out of smaller representations each one of which must be faithful to reality. If such a representation is expressed in terms of one or more representation languages, then at least two requirements must be met for all assertions that are part of the representation: (1) the symbols intended to denote entities denote only entities that exist or have existed and (2) the symbols which express relationships between these entities do so equally veridically, i.e. all posited relationships must obtain in reality. This means that the ontology must have a very precisely defined ontological commitment that is anchored in an ontological theory based on one or other form of realism. A requirement for high-quality ontologies – whether realism-based or not – is that the veridicality of all assertions made therein is verifiable. When part of the representation is expressed in a logical language, then the logical coherence and consistency of that part can be checked algorithmically. This is because logical connectors and quantifiers, if any at all, of such languages are precisely defined, as well as how they may be combined and what sorts of operations and transformations can be applied to them. Both of these requirements are satisfied by the Basic Formal Ontology (BFO): it is a domain-independent upper ontology [6] which is based on Ontological Realism [7]. Its most recent version, BFO2020-FOL, has recently been accepted as an ISO standard [8]. This includes an axiomatization in First Order Logic (FOL) for a fair part of its underlying philosophical principles and theories, enough to allow for sound spatial and temporal reasoning on top of the mere classification as offered by description logics.

## 3. Use case: history of cholangiocarcinoma in ‘relatives’

Cholangiocarcinoma (CCA) is a significant public health problem in Thailand, especially in the northeastern region [9, 10]. CCA is a lethal bile duct cancer which in countries of Southeast Asia is associated with *Opisthorchis viverrini* (OV) infection [11]. Roughly 5,000 new cases of CCA are diagnosed annually, and at least 8 million people are infected with OV in Thailand. In 2013, the Khon Kaen University (KKU) developed a prospective cohort study called the Cholangiocarcinoma Screening and Care Program (CASCAP) to eliminate OV and CCA. This led in collaboration with the National Health Security Office and the Ministry of Public Health to a national policy to improve diagnosis and treatment for CCA, covering all primary, secondary, and tertiary cares [12]. Subjects enter the CASCAP program in one of two ways [13]. One is through screening performed in high-risk areas on the basis of voluntary enrollment. This includes a structured interview followed by an ultrasound screening for CCA. Patients suspected of having a CCA may obtain a confirmation diagnosis through Computerized Tomography (CT) or magnetic resonance imaging (MRI) [14]. Subjects can also enter when they are diagnosed as having a CCA in hospitals from the CASCAP network.

The CASCAP administration maintains a data repository about subjects exhibiting the following inclusion criteria: (1) living in northeastern Thailand, (2) being at least 40 years old, and (3) either of the following: (3a) ever having been infected with or treated for liver fluke, or (3b) ever having consumed raw freshwater fish with scales. Data in the repository is collected by means of six forms [15]. One of them is the Demographic Information and Enrollment form CCA-01, which researchers and public health officers use to register participants and collect from them demographic information as well as certain risk factors for CCA, amongst which is having a family history of CCA [16, 17]. It is the latter topic that inspired us as a demonstration use case for ontology re-use and adaptation in line with realism-based principles. Family history is in the CCA-01 form determined by means of a yes/no response to the question ‘*Do you have any relatives diagnosed with cholangiocarcinoma?*’. When this question is positively answered, the following options are offered as categories: (1) paternal grandfather or mother, (2) maternal grandfather or mother, (3) older aunt or uncle, (4) younger aunt or uncle, (5) father or mother, (6) son or daughter, (7) brother or sister, (8) nephew or niece, and (9) spouse. In this paper, we discuss the development methodology of our kinship ontology as well as the challenges encountered. In a companion paper, we elaborate on how to use it for quality control of the CASCAP data-repository.

## 4. Existing kinship ontologies

Chui et al. propose a kinship ontology that they call ‘*T*_*kinship*_’ [18]. The ontology is axiomatized in First Order Logic (FOL) and consists of 13 axioms. Axioms (1)–(8) are concerned with *natural*-*ancestor-of*, (9)–(12) with *spouse-of*, and axiom 13 with both. Chui et al. use the phrase ‘ancestor’ rather than the more specific term ‘natural ancestor’ and in general do not append ‘natural’ to terms that admit of ‘non-natural’ readings (e.g., ‘grandparent’), but it is clear that when they use such terms they mean them to carry their blood-relative senses. Chui et al. also elaborate on how they think their axioms can be exploited so as to define some additional familial relations, such as the *has-natural-grandparent* relation.

Stevens et al. propose a different kinship ontology, the Family History Knowledge Database (FHKD), written in OWL 2 DL [19]. The FHKD was designed as a way of demonstrating OWL 2’s features and testing automated reasoners. As they admit, some of the axioms in their ontology, if interpreted as genuine claims about reality, are seriously questionable; for example, some of their axioms concerning *siblinghood* imply that a given person, S, is sibling of S [19, p5]. But in light of the educational and testing purposes for which the FHKD was designed, it seems charitable to interpret such axioms not as genuine claims about reality. The work also demonstrates that even the expressive DL SROIQ(D) is not expressive enough to represent kinship.

KIN, a DL kinship ontology produced as a part of the Global Alliance for Genomics and Health Pedigree Standard project [20], contains a fairly small number of defined classes, with *person* and *sex* subsuming the rest, and a larger hierarchy of object properties, at the top of which are hasSex and isRelativeOf, and further down relations such as isGestationalCarrierOf and isMitochondrialDonorOf. KIN is said to allow using an OWL reasoner to automatically validate a family history graph and infer new relations, while the expansiveness of its object properties would allow for detailed descriptions and inferences concerning individuals. However, like FHKD, KIN contains some axioms that yield implausible results if interpreted as genuine claims about reality. For example, KIN holds that isRelativeOf is symmetric and transitive, thus implying (implausibly) that a given person, S, is relative of S. Furthermore, KIN’s sex class-hierarchy is a bit peculiar. In addition to Female and Male, KIN contains OtherSex and UnknownSex, but OtherSex is a *subclass* of *Female or Male* (implying that it is *not* another sex, as might be had by some organism of an imaginable sexually ternary species); and KIN specifies that OtherSex is meant to cover cases in which ‘*It is not possible to accurately assess the applicability of male or female*’ (making one wonder what its difference from UnknownSex is supposed to be) [21].

Cantone et al. present several DL kinship ontologies, some for SROIQ and one for EL++ [22]. They explicitly note (as mentioned above) that FHKB treats isSiblingOf as reflexive, and they appear to treat avoidance of such results as a side-constraint on the development of an acceptable kinship ontology. Among their goals, however, are to show some of the limitations of SROIQ and EL++, by showing that the SROIQ and EL++ ontologies that they consider are logically weaker than a different plausible set of kinship axioms that they call ‘*K*_*L*_.’ For example, *K*_*L*_ contains an axiom to the following effect: x is relative of y iff (a) x and y are non-identical and (b) there is a sequence x…y every member of which with an immediate successor is relative of its immediate successor. As Cantone et al. point out, this axiom allows *K*_*L*_ to yield inferences that are unavailable to SROIQ and EL++ ontologies.

Although not itself a kinship ontology, SNOMED CT has a large number of kinship concepts, which are not classified as relations – in SNOMED CT called ‘attributes’ – but as ‘person’, a subhierarchy of ‘social entity’. In what follows, we reference SNOMED CT concepts through the concatenation of ‘sct_’, the fully specified name (with the first letter of the fully specified name capitalized as in SNOMED CT itself and spaces replaced by hyphens), a second underscore, and finally the concept’s semantic tag. Two broad classes of kinship concepts are worth noting. First, there is the concept sct_Blood-relative_person and all the concepts falling under it. These concepts, unsurprisingly, specifically correspond to *blood-relative* categories of which individuals can be members. Examples include, in addition to sct_Blood-relative_person itself, sct_Natural-sibling_person and sct_Natural-child_person. Second, there are familial concepts in SNOMED CT that do *not* specifically correspond to blood-relative categories. Examples include sct_Niece_person and sct_Maternal-grandparent_person; one can be a niece or maternal grandparent of someone else without being a blood relative of that person.

## 5. Methodology

We built our ontology following a number of steps, thereby reiterating over previous steps whenever deemed necessary. These steps were taken to satisfy the following requirements for our ontology: (1) maximally re-use what is available and be maximally re-usable itself, (2) be able to represent all kinship relations required for the CCA-01 form, (3) be fully BFO2020-compatible and (4) fit in the logical framework set up to combine realism-based ontologies with concept-based ontologies such as SNOMED CT [23].

Step 1 consisted of manually inspecting the axioms in the existing kinship ontologies to assess the degree to which they can be read literally, i.e. the extent to which they are faithful to the aspects of reality to which they pertain. For example, consider axiom (A1) from the *T*_*kinship*_ ontology:

(A1) ∀x(¬ancestorOf(x, x))

If we thought there were counterexamples to this axiom – involving backwards time-travel and causal loops or some other such exotic phenomena – then we would not import it in our ontology. This step included the identification of axioms that are most often satisfied, yet not in general. An example is axiom (A2) as found in *T*_*kinship*_.


(A2) $$\forall \text{x}\forall \text{y }\left(\text{hasChild(x,y)}\equiv \left(\text{ancestorOf}(\text{x},\text{ y})\wedge \neg \left(\exists \text{z}\left(\text{ancestorOf}(\text{x},\text{ z})\wedge \text{ancestorOf}(\text{z},\text{ y})\right)\right)\right)\right)$$

The sort of situation that counterexamples (A2) is indeed highly unusual: people do not ordinarily have natural children who are natural descendants of their own natural descendants. Readers familiar with the classic 1974 film *Chinatown* might recall that one of the big revelations toward the end of the movie is that one of the characters had fathered a child with his own daughter. This would be a situation of the sort at issue. Such axioms were however not excluded from our ontology but turned into related axioms in such a way as to mark the peculiarity of the situation.

Step 2 was to rewrite the accepted axioms so as to be fully compatible with the BFO2020-FOL axiomatization. Several transformations were to be considered. One was to rewrite predicates which for BFO are considered ‘*fantologically conceived*’ [24]. Examples appear in axiom (A3) from *T*_*kinship*_.


(A3) $$\forall \text{x}\forall \text{y}\left(\text{ancestorOf(x,y)}\to \left(\text{person}(\text{x})\&\text{person}(\text{y})\right)\right)$$

In FOL as used in *T*_*kinship*_, ‘ancestorOf’ and ‘person’ are relations – binary and unary resp. – that hold for certain individuals in the domain of discourse. FOL allows one to predicate something about individuals in its domain without being bothered by any ontological commitment. While BFO might commit to ‘ancestorOf’ representing an instance-level formal relation [25], it would not commit to ‘person’ representing a formal relation but rather a universal instantiated by а particular at a time. A BFO-compatible FOL translation of (A3) would therefore be:

(A4) $$\forall \text{x}\forall \text{y}\left(\text{ancestorOf(x,y)}\to \left(\exists \text{t}1\exists \text{t}2\left(\left(\text{instance-of}\left(\text{x},\text{ person, t}1\right)\&\text{instance-of}\left(\text{y},\text{ person, t}2\right)\right)\right)\right)\right)$$

In general, any axiom in a source ontology that describes an individual as timelessly standing in some unary relation (as in FOL ‘*fantologically conceived*’ [24]) or as timelessly being a member of a class (as in OWL) required revision for BFO2020-compatibility during Step 2. This is because under a realism-based perspective, such an individual is very likely a particular which instantiates at a time a universal. Such revision was also needed for cases expressing some individual’s timelessly standing in some relation to some class member when such а relation would require time-indexing as per BFO’s ontological commitment.

Step 3 consisted of replacing terms for relations and universals with terms that express better what is intended. Examples included replacing occurrences of ‘ancestorOf’ with occurrences of ‘natural-ancestor-of’ to make explicit that our axioms containing this expression are concerned with natural ancestry and not with some broader notion of ancestry, and replacing occurrences of ‘person’ with occurrences of ‘human-being’.

Step 4 consisted in formulating axioms detailing relations between the kinship relations referenced on the CCA-01 form to the kinship relations completed thus far. Only one relation referenced on the form, the *has-spouse* relation, we did not address at this stage, because it already appeared in our collection. The other relations referenced on the form are all blood relations, despite the fact that the English version of this form confusingly contains some non–blood-relation-specific terms such as ‘son’, ‘daughter’, and ‘sibling’; the Thai terms carry specifically blood-relation senses only.

We devised in Step 5 bridging axioms from the relations and universals referenced in the axioms produced thus far to corresponding kinship terms found in SNOMED CT and vice versa, as exemplified by (A6) and (A5) respectively. Although many of these axiom pairs can be written as biconditionals, we refrained from doing so for reason of modularization towards re-usability on a needs basis.


(A5) $$\forall \text{x}\left(\text{individual-of}\left(\text{x},\text{ sct\_Natural-child\_person}\right)\to \left(\exists \text{y}\left(\text{has-natural-child}\left(\text{y},\text{ x}\right)\right)\right)\right)$$

(A6) $$\forall \text{x}\left(\left(\exists \text{y}\left(\text{has-natural-child}\left(\text{y},\text{ x}\right)\right)\right)\to \text{individual-of}\left(\text{x},\text{ sct\_Natural-child\_person}\right)\right)$$

In Step 6, we devised a set of axioms specifying certain highly unusual ancestry and spousal situations as unusual. These axioms are inspired by axioms from *T*_*kinship*_ that we have rejected because they admit of counterexamples. An example is the rejected axiom (A2) (discussed above), from which we derived (A7).


(A7) $$\forall \text{x}\forall \text{y}\forall \text{z}\left(\left(\text{has-natural-child}\left(\text{x},\text{ y}\right)\&\text{natural-ancestor-of}\left(\text{x},\text{ z}\right)\&\text{natural-ancestor-of}\left(\text{z},\text{ y}\right)\right)\to \text{occupy-unusual-ancestry-situation}\left(\text{x},\text{ y},\text{ z}\right)\right)$$

We note that we do not mean by ‘unusual’ ‘counterintuitive’. For example, a spousehood relation between first cousins is not, in our view, counterintuitive, though we mark such a relation as unusual. A better gloss on ‘unusual’ is ‘atypical’. Spousehood relations between first cousins are atypical—they just don’t happen very often. Furthermore, they happen sufficiently infrequently that an unusualness axiom pertaining to them seems to serve valuable data-entry and -inspection purposes, as we explain in Section 6. We also emphasize that ‘unusual’, as we use it, is not meant to carry any normative weight. In calling a relation ‘unusual’, we do not mean to imply that it is bad, that it ought to be illegal, or that any other such normative fact obtains.

As a last step, we translated all axioms in CLIF following the schema of the BFO2020-FOL axiomatization in CLIF, used a parser-generator to transform the axiom collection in a Kowalski-rule base, and the latter as input for a reasoner for satisfiability testing [26]. Kowalski rules are a further transformation of FOL axioms after they have been translated into clausal normal form. Kowalski rules are logical implications of which the antecedent is formed by conjoining the atoms of the negative literals in a clause, and the consequent from the disjunction of the positive literals [27].

## 6. Results

Our kinship ontology consists of six modules. Some modules contain axioms whose definientia refer to relations or entities defined in other modules. However, some modules can be ignored when irrelevant for certain applications. That is for instance the case for the bridging axioms to and from SNOMED CT when SNOMED CT is not used in an application the ontology intends to serve.

Each axiom comes with a short textual description terminated by an index which is unique within and across all modules. This index can be used to import individual axioms as well as to create additional documentation containing detailed textual definitions and elucidations. It can also be used to link the ontology to a terminology.

The core module, ancestry.clif, consists of 56 axioms, each one of which belongs to one of the following categories: (1) replacements of the *T*_*kinship*_ axioms, subindexed ‘tkr’; (2) axioms for ordinary kinship relations requested on the CCA-01 form, subindexed ‘cca’; (3) a recursive definition of the natural-ancestor-of relation, split into two axioms each subindexed ‘nao’; (4) additional axioms for ordinary kinship relations, subindexed ‘ak’; and (5) axioms linking universals referenced in this module to BFO categories, subindexed ‘u’. Most of these relations are fairly ‘ordinary’ ones, such as the *natural-maternal-grandparent-of* relation and the *natural-sibling-of* relation: they are ordinary in the sense that they are to be interpreted as literal in all contexts. But a few ‘extraordinary’ relations, not commonly treated in kinship ontologies, appear on the CCA-01 form as well, including the *natural-older-uncle-of* relation. The Thai expression which appears on CCA-01 for this relation refers to a person who is an older biological brother of one of one’s biological parents. These and other unusual relations we axiomatized in the separate module, cca01-ground.clif. The axioms therein are also to be interpreted literally – i.e. they represent universal ground truth – but are not useful in an environment in which such relations are not considered.

Three modules provide a bridge to and from SNOMED CT. Axioms linking from relations and universals to SNOMED CT concepts are in ancestry-sct.clif; those linking from SNOMED CT concepts to relations and universals are in sct-ancestry.clif. For example, ancestry-sct.clif contains an axiom to the effect that if x is natural parent of someone, then x is an individual of sct_Natural-parent_person; and sct-ancestry.clif contains an axiom to the effect that if x is an individual of sct_Natural-parent_person, then x is natural parent of someone. Whenever one of these modules is imported, the axioms in sct-declaration.clif also need to be imported. This module contains a collection of axioms, the first member of which states that if x is an individual of y, then x is a particular and y is a class, and the other members of which state about each term taken from SNOMED CT that it is a class. For example, one of the axioms in sct-declarations.clif states that sct_Natural-parent_person is a class.

The module unusual.clif contains 4 axioms pertaining to highly unusual situations, in which (1) a person z has a natural descendant which has as parent a natural ancestor of z; (2) a pair of close blood relatives are co-natural-parents of someone; (3) a pair of close blood relatives are spouses; and (4) some people are in a plural spousal situation. These unusual-case axioms can serve valuable data-checking purposes. If someone enters into an electronic medical record data that trigger one of these axioms (by, say, entering data to the effect that Fred and Sally are spouses while a contemporaneous spousal relation between Fred and Catherine is already on record), then a warning message can be devised recommending that the entered data be double-checked for accuracy. Given how unusual the situations in question are, the triggering data-entry will often have been erroneous.

For the sake of convenience, we also compiled a file, inspiration.clif, containing *T*_*kinship*_ translated into CLIF but otherwise left untouched. This file is not to be considered part of our kinship ontology.

All axiom files as well as certain additional documentation is available via the following link: https://buffalo.box.com/s/pn9rv6m0i7wkcfow48f9270c4jh3kp6c

## 7. Discussion

Crucial to our ontology is the *has-natural-child* relation; the *natural-ancestor-of* relation is defined recursively partly in terms of it. Other blood relations are defined in our ontology partly in terms of one or the other of these two relations. By ‘partly’, we mean that additional information is needed to fully grasp the intended meaning. Although such information can be axiomatized as well, and should be done for other purposes extending kinship between individuals, it would not lead to useful reasoning in the context of CASCAP. By ‘has-natural-child(Amy, Bob)’, for example, we mean that among the gametes from which Bob originates is a gamete of Amy. By ‘of Amy’ in this context, we do not mean (say) *owned by Amy* or *controlled by Amy*, but rather *having its biological origin in Amy*. If Amy has sold one of her ova, O, to Clair, then O is in a loose sense a gamete *of Clair* but is not *of Clair* in the sense relevant here. The expression ‘natural’ is thus to be understood throughout our ontology as helping to designate blood relations. We use the term ‘natural’ in this context, as opposed to (say) ‘biological’ or ‘blood’, simply because ‘natural’ is also the term used in most SNOMED CT blood-relation concepts, and relevant modules in our ontology function as a bridge between SNOMED CT and BFO.

Many relations in our ontology, as in many kinship ontologies, are also partly defined in terms of sexes. In our axioms, the expression ‘male-sex’ picks out the sex had by males qua males, and ‘female-sex’ picks out the sex had by females qua females. We insist that ‘male-sex’ picks out the sex had by males qua males, not the sex had by males simpliciter (and similarly, mutatis mutandis, for ‘female-sex’ and females). Simultaneous hermaphrodites (e.g., great pond snails) are male and female at the same time. It follows that there is no such thing as the sex had by males simpliciter, for some males have multiple sexes. But even a simultaneous hermaphrodite has the male sex, and only the male sex, qua male. Hence our insistence on the “qua” restriction.

We also use the term ‘spouse’ to pick out a *marriage-partner*, and the expression ‘has-spouse(x,y)’ to mean that x has a marriage partnership with y. We take a rather minimal stand on the metaphysics of marriage – concerning who can enter into it, how many individuals can enter into a given marriage, whether marriage is ‘purely legal’ and so on. Our stand is not utterly neutral, though. For example, one axiom of ours entails that the *has-spouse* relation is irreflexive: you can’t be married to yourself. We also assume that if x has spouse y, then there is a marriage bond that inheres in x and y and that exists at a time at which x and y exist. This assumption secures the intuitively correct verdict that spousehood is a *temporal* matter; people are not simply atemporally spouses of one another, even if natural-ancestry relations are simply atemporal. At present, our ontology says nothing about other non-blood relations beyond spousehood, such as domestic partnerships, close friendships, and so on; though it could be extended to comprehend such relations.

### 7.1. Time indexing

Because time-indexing plays such an extremely important role in BFO, a package of questions guiding our project was which elements of our ontology required time-indexing, which did not, and how the appropriate time-indexings would be best accomplished. The axioms in the ontologies discussed in Section 4, including *T*_*kinship*,_ treat (say) *being a person* as a matter of timelessly bearing some property, or as a matter of timelessly being, or being related in some way, to a member of some class. One feature of our ontology that makes it different from those discussed in Section 4 is thus that all such talk is replaced by talk of *individuals’ being instances of universals at times*, as in, for example, the following axiom:

(A8) $$\forall \text{x}\forall \text{y}\left(\text{natural-father-of}\left(\text{x},\text{ y}\right)\equiv \left(\text{natural-parent-of}\left(\text{x},\text{ y}\right)\&\exists \text{q}\exists \text{t}\left(\text{instance-of}\left(\text{q},\text{ male-sec},\text{ t}\right)\&\text{inheres-in}\left(\text{q},\text{ x}\right)\right)\right)\right)$$

Relations between individuals included in our ontology were a somewhat trickier matter than universals, because for some of these relations time-indexing seems appropriate but for others it does not. For example, it is plausible that *natural-ancestor-of* is non–time-indexed: it seems atemporally true that, for example, Abraham Lincoln’s maternal grandfather is among Lincoln’s ancestors. By contrast, consider *has-spouse*. Some form of time-indexing seems appropriate for this relation, for people are married to one another for specific time periods, and a given person can be married to different people at different times. One way to accommodate an element of time-indexing concerning *has-spouse* is to time-index *has-spouse* itself; another is to attach time-indexing to something that one’s ontology holds to be inextricable from spousal relations. As mentioned above, we took the latter approach, by maintaining that x has spouse y if and only if there is a marriage bond – a specialization of BFO2020’s relational quality – that exists *when x and y do* and that inheres in x and y.

### 7.2. Bridging to SNOMED CT

The meaning of each SNOMED CT term is provided either through an individual concept or by at least one axiom expressed in the description logic EL++ [28]. Some of us have described elsewhere some of the potential benefits of using bridge axioms to attach the terminological richness of SNOMED CT to the ontological foundation supplied by BFO [23, 29]. The approach defended in [23, 29] is to let SNOMED CT’s view and BFO’s view happily co-exist, not in one *ontological* framework, but in one *logical* model-theoretic framework capable of exploiting what SNOMED CT offers *terminologically* and realism-based ontologies offer *ontologically*. We developed the modules sct-ancestry.clif, ancestry-sct.clif and sct-declarations.clif of our kinship ontology partly as a proof of concept of this general bridging strategy.

While devising our ontology, we encountered a number of challenges. For example, some of the relations at issue in ancestry.clif and cca01-ground.clif do not have precisely corresponding SNOMED CT concepts. For instance, one relation defined in cca01-ground.clif is the *natural-older-uncle-of* relation. Unsurprisingly, sct_Natural-older-uncle_person does not exist in the international version of SNOMED CT. Perhaps a bit more surprisingly, there is also no SNOMED CT concept precisely corresponding to the *natural-ancestor-of* relation. In these cases, bridging axioms cannot be proposed. When, however, a relation in our ontology lacked a precisely corresponding SNOMED CT concept, but there was a SNOMED CT concept that *nearly* precisely corresponded to the relation and a true bridge axiom connecting them could be imagined, we chose to include such an axiom. For example, because *natural-niece-of* and sct_Niece_person nearly precisely correspond and sct_Natural-niece_person does not exist, we included in our ontology the following bridging axiom:

(A9) $$\forall \text{x}\left(\exists \text{y}\left(\text{natural-niece-of}\left(\text{x},\text{ y}\right)\right)\to \text{individual-of}\left(\text{x},\text{ sct\_Niece\_person}\right)\right)$$

A related issue concerned the question when bridging axioms could be formulated in both directions and when they could not be. In situations involving precisely corresponding kinship relations and SNOMED CT concepts, axioms in both directions were warranted; in situations not involving such precise correspondence, this was not the case. Hence the axioms (A10) and (A11), for example, both appear in our ontology, because *natural-sibling-of* and sct_Natural-sibling_person precisely correspond:

(A10) $$\forall \text{x}\left(\exists \text{y}\left(\text{natural-sibling-of}\left(\text{x},\text{ y}\right)\right)\to \text{individual-of}\left(\text{x},\text{ sct\_Natural-sibling\_person}\right)\right)$$

(A11) $$\forall \text{x}\left(\text{individual-of}\left(\text{x},\text{ sct\_Natural-sibling\_person}\right)\to \exists \text{y}\left(\text{natural-sibling-of}\left(\text{x},\text{ y}\right)\right)\right)$$

By contrast, the right-to-left analogue of (A9) does not appear in our ontology.

At present, our kinship ontology focuses primarily (though not exclusively) on blood-relations, and we have accordingly focused up to now on linking SNOMED CT blood-relative concepts to our ontology and proposing axioms defining relations corresponding to such concepts. However, there are a great many SNOMED CT blood-relative concepts, some of them highly specific, and we have not thus far tried to link every SNOMED CT blood-relative concept to our ontology or to propose axioms defining relations corresponding to every such concept. For example, sct_Identical-twin-brother_person falls under sct_Blood-relative_person, but we have not yet linked this concept to our ontology, we have not proposed an axiom defining the identical-twin-brother-of relation, and so on. Still, the work we have done thus far could be extended so as to cover all SNOMED CT blood-relative concepts, and indeed we hope in future work to do that.

### 7.3. Unusual kinship relations

A different stage of our project that required judgment calls on our part was the production of unusual.clif. Consider, for example, the following axiom in unusual.clif:

(A12) $$\forall \text{x}\forall \text{y}\forall \text{z}\left(\left(\text{has-natural-child}\left(\text{x},\text{ z}\right)\&\text{has-natural-child}\left(\text{y},\text{ z}\right)\&\left(\text{natural-parent-of}\left(\text{x},\text{ y}\right)\vee \text{natural-sibling-of}\left(\text{x},\text{ y}\right)\vee \text{natural-grandparent-of}\left(\text{x},\text{ y}\right)\vee \text{natural-aunt-of}\left(\text{y},\text{ x}\right)\vee \text{natural-uncle-of}\left(\text{y},\text{ x}\right)\vee \text{natural-first-cousin-of}\left(\text{x},\text{ y}\right)\right)\right)\to \text{occupy-unusual-ancestry-situation}\left(\text{x},\text{ y},\text{ z}\right)\right)$$

The intuitive idea behind (A12) is that if two people are co-natural-parents of a common person and are themselves close blood relatives, then they and their child occupy an unusual ancestry situation. The long disjunctive clause specifies a range of blood-relations that clearly qualify as *close*. But, of course, there are other close relations not covered by the clause (e.g., *natural-second-cousin-of*). Adding additional such relations to the disjunctive clause would allow for the generation of additional data-entry warnings, and so would probably flag some incorrect data-entries that would otherwise go undetected. However, expanding the disjunctive clause in certain imaginable ways might also generate enough warnings in cases of *correct* data-entry that doing so would not be all-things-considered prudent. For example, if one were to revise (A12) in such a way that co-natural-parenting natural seventh cousins count as occupying an atypical ancestry situation, then one would perhaps thereby revise (A12) in such a way that it yielded a counterproductively large number of false positives.

We suggest that (A12) be read as an *example* of a warning-case axiom relevant to the situations to which it pertains, not as *the best possible version* of such an axiom. This axiom and the others in unusual.clif could be revised to suit particular data-entry contexts, or even rejected altogether. For example, the axiom (A13) is in unusual.clif, we hope for obvious reasons:

(A13) $$\forall \text{x}\forall \text{y}\forall \text{z}\left(\exists \text{t}\exists \text{m}1\exists \text{m}2\left(\text{instance-of}\left(\text{m}1,\text{ marriage-bond},\text{ t}\right)\&\text{instance-of}\left(\text{m}2,\text{ marriage-bond},\text{ t}\right)\&\text{inheres-in}\left(\text{m}1,\text{ x}\right)\&\text{inheres-in}\left(\text{m}1,\text{ y}\right)\&\text{inheres-in}\left(\text{m}2,\text{ x}\right)\&\text{inheres-in}\left(\text{m}2,\text{ z}\right)\&\sim \text{y}=\text{z}\right)\to \text{occupy-unusual-spousal-situation}\left(\text{x},\text{ y},\text{ z}\right)\right)$$

However, if one lives in a polygamous society, then it might be a good idea for one not to adopt axiom (A13) at all. Though even this is not obviously right: if one lives in a society in which some but very few people practice polygamy, then (A13) might be worth adopting after all.

## 8. Conclusion

We have developed a novel kinship ontology in First Order Logic following the representational principles of BFO2020-FOL. The ontology comes in separate CLIF-modules each one of which can be imported based on specific needs, for example, mapping to and from SNOMED CT, or exploiting axioms which would not be literally true when phrased naively but are crafted in a way that allows the generation of alerts on possible data entry mistakes. The ontology can be used directly by CLIF-reasoners, or translated into much weaker versions of the axioms for OWL-DL reasoners. In future work, we intend to expand on this project by adding SNOMED CT kinship concepts that we have not yet wedded to our kinship ontology, either through definitions of corresponding relations or through production of relevant bridge axioms. Further expansion following the same bridging strategy might happen when other relevant kinship terminologies become prominently used.
